# A Method of Interstory Drift Monitoring Using a Smartphone and a Laser Device

**DOI:** 10.3390/s20061777

**Published:** 2020-03-23

**Authors:** Jinke Li, Botao Xie, Xuefeng Zhao

**Affiliations:** State Key Laboratory of Coastal and Offshore Engineering, School of Civil Engineering; Dalian University of Technology, Dalian 116024, China; jinkeli@mail.dlut.edu.cn (J.L.); botaoxie@mail.dlut.edu.cn (B.X.)

**Keywords:** seismic monitoring, smartphone, interstory drift, laser device, structural health monitoring

## Abstract

Interstory drift is an important engineering parameter in building design and building structural health monitoring. However, many problems exist in current interstory drift monitoring methods. The traditional method is imprecise—double numerical integration of acceleration data—and other direct monitoring methods need professional equipment. This paper proposes a method to solve these problems by monitoring the interstory drift with a smartphone and a laser device. In this method, a laser device is installed on the ceiling while a smartphone is fixed on a steel projection plate on the floor. Compared with a reference sensor, the method designed in this study shows that a smartphone is competent in monitoring the interstory drift. This method utilizes a smartphone application (APP) named D-Viewer to implement monitoring and data storage just in one place, which is also inexpensive. The results showed that this method has an average percent error of 3.37%, with a standard deviation of 2.67%. With the popularization of the smartphone, this method is promising in acquiring large amounts of data, which will be significant for building assessment after an earthquake.

## 1. Introduction

When an earthquake occurs, seismic wave propagation will cause translation and rotation of building structure [1], which has caused countless loss of lives and has affected the economy. In order to improve the capacity of the structure and reduce losses, many codes set criteria on the engineering parameters of buildings. Interstory drift is a widely used parameter among these engineering parameters, which is defined as a relative displacement between two consecutive floors. Interstory drift could be normalized by story height, then it turns into an interstory drift ratio and often used in codes. For example, HAZUS, developed by the Federal Emergency Management Agency (FEMA) [2], stipulates different damage levels with different interstory drift values based on the structure type. According to the Code for Seismic Design of Buildings [3], a building’s deformation, such as interstory drift, should meet the requirements of the fortification against earthquakes. FEMA 356 [4] categorizes target building performance level into four classes: operational level, immediate occupancy level, life safety level, and collapse prevention level. The corresponding limits of maximum interstory drift ratios are set based on different structure types.

Apart from these codes, interstory drift is widely used in experiments [5,6,7], simulations, and damage detection [8,9,10]. As a common engineering parameter, story shear versus interstory drift is often used to indicate structure stiffness variation. For example, Suita et al. [5] used the pictures of story shear versus interstory drift ratio to represent lateral stiffness variation history, in the collapse experiment of a four-story full-scale steel moment-resisting frame on E-Defense. Okazaki et al. [6] also used this kind of model to show the nonlinear behavior of concentrically braced frames. Zhou et al. [7] extracted half cycles of hysteresis loops in this picture as a histogram of stiffness features and used this feature to build a deep learning network in order to detect building structure damage. Besides, there are also numerous applications of using interstory drift as a damage index or part of the damage index. Through the training of an Artificial Neural Networks model, Morfidis and Kostinakis [8] suggested that at least five seismic parameters should be imported into the model to predict the maximum interstory drift ratio of a reinforced concrete (RC) building, and this maximum interstory drift ratio was chosen as the only damage index. In order to assess the structural damage of two experiments [5,6], Hwang et al. [9] built a connection between wavelet-based damage-sensitive features and peak interstory drift ratios, and tried to quantify this wavelet-based damage indicator. Xiang et al. [10] only measured the interstory drift in experiments conducted on E-Defense and used this engineering parameter to construct residual drift and plastic deformation ratios, which were used to evaluate beam damage. 

The examples mentioned earlier, demonstrate interstory drift is a significant engineering parameter in building design, experiment, simulation, and damage assessment. However, due to the cost and other reasons, interstory drift monitoring is staying in the laboratory, and not popularized in actual buildings so far. 

The essence of interstory drift monitoring is to monitor relative displacement between ceiling and floor. There are many relative displacement monitoring methods, such as a linear variable differential transmitter (LVDT) [11], double numerical integration of acceleration data [11,12,13,14], the photogrammetry-assisted Augmented Reality (AR) technique [15], Micro Electro Mechanical Systems (MEMS) inclinometers [16], and the machine vision method [17]. 

These applications will be introduced in detail. In order to measure the aftershock response of 4 RC buildings after the 8.8 Maule earthquake in Chile, Lemnitzer et al. [12] installed accelerometers and some LVDTs in these buildings and monitored them for a month. The interstory drift was also used in building assessment, which was identified through double-integrating the accelerations by applying a high-pass filter. Spina et al. [13] introduced the Seismic Observatory of Structures (OSS) system in Italy to monitor the dynamic response of 128 buildings. This OSS system could generate the reports, in which the interstory drift was calculated by double numerical integration of acceleration data. Dai et al. [15] proposed a photogrammetry assisted AR technique using a consumer-grade digital SLR camera to measure interstory drift, which could be used after seismic events. Hou et al. [16] calculated the relationship between interstory drift ratio and rotation of a column in both elastic and elastoplastic stages and used 8 MEMS inclinometers in one column to verify the feasibility of the method. The results turned out that the mean percent error was 7.6%. Li et al. [17] used a smartphone that was fixed on the floor with the front camera recording video of the ceiling. A feature point-matching algorithm was used to recognize ceiling objects and calculate the relative movement between the ceiling and floor. A more comprehensive collection of different kinds of technologies used in relative displacement monitoring is presented in two literature reviews [11,18]. 

However, the above methods have disadvantages. For example, an extra high rigid support will be required to install LVDT. Influenced by electrical and mechanical hysteretic effects, cross-axis effects, et al. [11], double numerical integration of acceleration data is imprecise. The most common error of this method is the baseline shift. Although there are many methods like baseline correction, band-pass filtering, et al. [11,14] to eliminates errors, subjective selection of filter type and cutoff frequency will still bring error to the results [11]. The photogrammetry assisted AR technique [15] is a static measurement, and it had not been tested in full-scale experiments. Using inclinometers [16] to monitoring interstory drift will require eight sensors in one column, which will increase the cost. Besides, the machine vision method [17] needs normal light and cannot be used in darkness.

In addition to the above-mentioned methods, the more accurate method is to monitor the interstory drift directly with two separate instruments installed on the ceiling and floor separately [19,20,21,22]. Kanekawa et al. [19] installed a laser light source on the ceiling and a relative displacement sensor composed of a photo scattering plate and a phototransistor (PT) array on the floor. When the photo scattering plate scattered the laser beam, the scattered light would illuminate two PT arrays, and then the laser beam center could be calculated and used to calculate interstory drift. Matsuya et al. [20] proposed a sensor system composed of three position-sensitive detectors (PSD) on the floor and three light-emitting diodes (LED) on the ceiling. Then, three engineering parameters of relative-story displacement, the local inclination angle, and the torsion angle could be measured simultaneously. Matsuya et al. [21] conducted a shaking table experiment in an actual building with a PSD unit on the ceiling and a LED on the floor to measure interstory drift time history, and this method had a high accuracy of 0.06 mm. Islam et al. [22] proposed a method that a laser sensor was mounted on the ceiling with a 45° inclined target surface fixed on the floor. The laser sensor could sense the distance change, and the interstory drift could be identified through simple calculations. McCallen et al. [23] used a laser source and a discrete diode position sensor (DDPS) to measure the interstory drift. They also proposed a method to correct the result caused by structure member rotation. Nevertheless, these methods require professional equipment, which will increase the cost. Thus, a low-cost interstory drift monitoring method needs to be explored.

Aiming to decrease monitoring costs and enable public participation, the application quantity of using smartphones in SHM is increasing fast. These applications include pedestrian link bridge vibration monitoring [24], in-plane displacement monitoring [25], three-dimension steel frame model damage detection [26], three-dimension displacement monitoring [27], building dynamic characteristics monitoring [28,29], bridge seismic monitoring [30]. Among these applications, the following three applications used smartphones to monitor displacement. Zhao et al. [25] used a smartphone and a laser point to monitor displacement in the laboratory and verify the feasibility of an app called D-Viewer. Xie et al. [26] used this method to record the displacement history of a shaking table test of a three-dimensional steel frame. Wang et al. [27] developed this method and made this App be able to monitor the out-of-plane displacement. However, the distances in these three examples between smartphones and objects were less than 400 mm. If this method could be used in interstory drift monitoring, it should satisfy a monitoring distance of a story height of 3000 mm [3]. 

From these examples, the challenges in interstory drift monitoring are precision, continuous measurement, full-scale experiment verification. Taking the challenges and above technique characteristics into account, this study is trying to propose an interstory drift monitoring method, which could fulfill these following requirements:

- The method should be available at a low cost.
- The method could guarantee continuously monitoring regardless of lighting changes.
- The measurement accuracy should below 0.1 mm, and measurement amplitude should be higher than ±50 mm. The percent error should be lower than 5%.
- The method should be feasible in a full-scale experiment.

In order to decrease costs, a smartphone combined with a laser device and a projection plate was used in this study. The smartphone is considered as the primary sensor, which integrates image processing, data acquisition, and data storage just in one place, and it is popularized. The usage of the laser device is to monitor at night without the influence of lighting changes. The experiments conducted in this study were under a story height 3000 mm, which will satisfy full-scale tests. Besides, the measurement accuracy will be verified in this study. The main contribution of the study is that the proposed method is cost-effective, and it has enough accuracy in interstory drift monitoring. 

The rest of the paper is organized as follows. First, the details of the methods are introduced, including the image processing principle, experiment setup, and D-Viewer app settings. Second, the results of three kinds of experiments—sinusoidal excitation, seismic wave excitation, and night test—are compared with reference sensors for verification and discussed. Third, the errors, feasibility, and cost of this method are discussed, as well as sensor comparison. Finally, the conclusions are drawn and future work is discussed. 

## 2. Materials and Methods

In this section, four parts closely related to the experiments will be introduced in order. These four parts include the image processing principle, the experiment setup, D-Viewer app settings, and the reference sensor. 

### 2.1. Image Processing Principle 

As a smartphone integrates cameras, it could be used as a sensor to shoot a picture or record videos. In this study, a smartphone was fixed parallel to a projection plate, so the relative movement of a laser spot could be recorded. Then, the video could be analyzed to identify the position of the laser spot in each frame. The following principles have been programmed into a smartphone application (app) called D-Viewer [25], and it was used in this study. 

**Figure 1:**
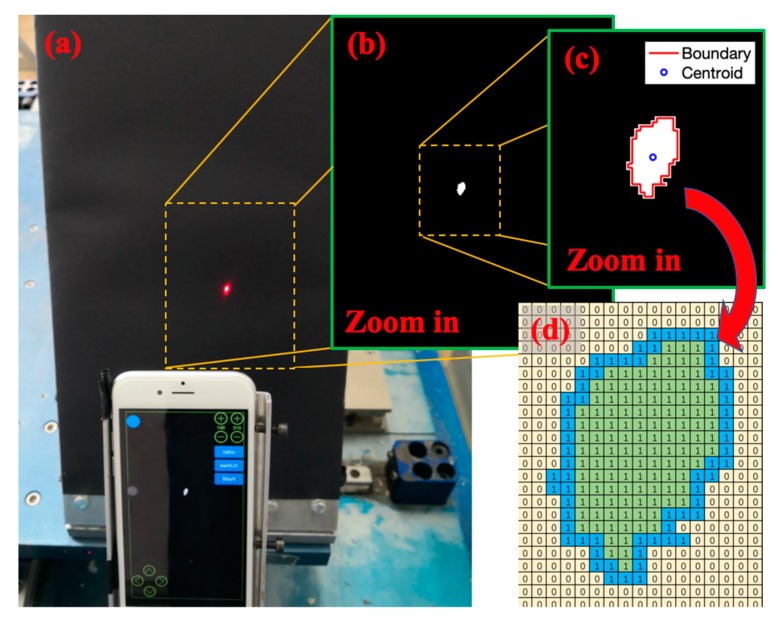
Image processing principle explanation: (**a**) Laser spot on a projection plate; (**b**) Gray processing and binarization processing; (**c**) Boundary detection and centroid calculation; (**d**) The value of the laser spot after binarization processing.

The illustration of the image processing principle is shown in Figure 1. The basic principle of the method could be divided into four parts: gray processing, binarization processing, boundary detection, and centroid calculation. 

In gray processing, the origin Red-Green-Blue (RGB) image in Figure 1a can be converted into a grayscale image by calculating the average of three components (R, G, B value). Because the intensity value of grayscale image has a range of 0~255, in the binarization processing, a set threshold value could make grayscale below this value equal to 0 and above this value equal to 1. As shown in Figure 1b, the laser spot is white after this process. 

Then it comes to boundary detection, a connected set of the laser spot should be detected firstly. Simple code could recognize the laser spot white circle as a connected set, and this connected set is denoted as region R1. The boundary of a region R1 (value = 1) is a set of points that are adjacent to points in the complement region R0 (value = 0) [31]. As shown in Figure 1d, the pixels of value 1 in the boundary are 8-adjacent [31] with pixels of value 0. As a result, the conditional statement loop code could be used to find the pixels in a boundary. 

At last, the centroid coordinates of the boundary are identified through Equation (1):

(1) $$\{\begin{array}{c} x_{c}=\frac{\sum_{1}^{N}x_{i}}{N}\\ y_{c}=\frac{\sum_{1}^{N}y_{j}}{N} \end{array}$$

where $\left(x_{c},y_{c}\right)$ are the pixel coordinates of the centroid, *N* is the total number of boundary pixels, $x_{i}$ and $y_{i}$ are ith pixel’s coordinates in the boundary. 

Through looping the process above, the laser spot coordinates in each frame could be identified. The displacements in the following frames are calculated through subtracting the first frame’s coordinates. 

### 2.2. Experimental Setup

When an earthquake strikes an area, due to the relatively high rigidity of the floor slab, the small inclination of the floor slab could be neglected [21]. Thus, the main movement of the building floor is horizontal. Therefore, the experiments should simulate the relative movement between the floor and ceiling. 

**Figure 2:**
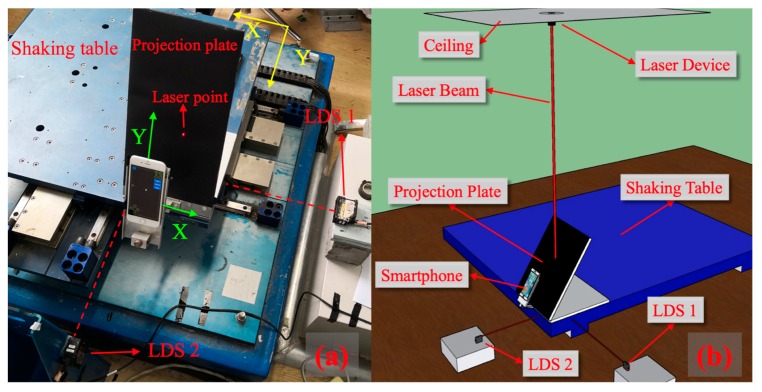
The layout of the experiments: (**a**) A close shot; (**b**) A schematic diagram.

Inspired by some of the referenced studies [19,20,21], a laser device was installed on the ceiling with a laser spot projected on the floor. If the movement of this laser spot could be recorded, the interstory drift could be identified. The shaking table test was conducted to simulate the relative movement between ceiling and floor (the floor represents the shaking table in this paper). Under this condition, using the method in paper [25], the laser spot’s movement was recorded through a smartphone camera. However, if the smartphone is parallel to the floor to shoot the laser spot on the floor, this laser spot might be sheltered by smartphone. As a result, a projection plate was designed to not shelter the laser spot. Meanwhile, the smartphone recorded the laser spot’s movement. In order to verify the method, laser displacement sensors (LDS) were used as a reference. The experiment scheme is shown in Figure 2.

**Figure 3:**
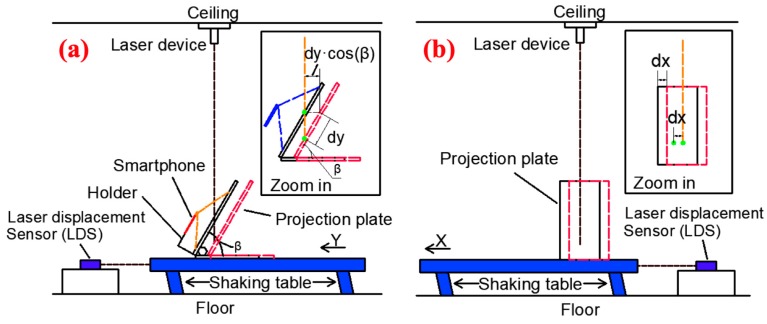
A schematic diagram of the experiment: (**a**) The elevation view (vibration direction along Y-axis); (**b**) The side view (vibration direction along X-axis).

The details of the experiment are introduced as follows. First, a laser device was installed on the ceiling, and its laser beam was perpendicular to the floor. A projection plate was composed of two steel plates and a smartphone holder, and the projection plate was fixed on the shaking table by bolts. As shown in Figure 3a, the angle between the projection plate and the shaking table was β (β=58° in the experiments). Besides, the smartphone holder ensured that the smartphone was parallel to the projection plate. The distance between the smartphone and projection plate was 150 mm, and this distance should be adjusted to ensure the movement of laser point within the recorded video. If the laser point moved out of the smartphone shooting range, displacement would not be recorded. Moreover, the frame in the recorded video should not beyond the edge of the projection plate because this will lead to the image processing algorithm failure. Third, the rear camera captured the image of the laser spot on the projection plate. 

As shown in Figure 3, the laser light path was not sheltered by the smartphone when the vibration was fierce. From two zoom-in pictures in Figure 3, the relationship between the movement on the floor of the laser spot and the movement on this projection plate could be identified through Equation (2):

(2) $$\{\begin{array}{c} D_{x}=d_{x}\\ D_{y}=d_{y}\cdot \cos\left(\beta \right) \end{array}$$

where $D_{x}$ and $D_{y}$ are the interstory drift in two directions, $d_{x}$ and $d_{y}$ are the displacements of the laser spot on the projection plate, β is the angle between the projection plate and the shaking table. 

An iPhone 6 was used in the experiments, and it was fixed on the holder of the projection plate. This iPhone 6 utilized its camera to record videos of the laser spot’s movement and calculate the displacement through its central processing unit (CPU). 

### 2.3. D-Viewer App Settings

**Figure 4:**
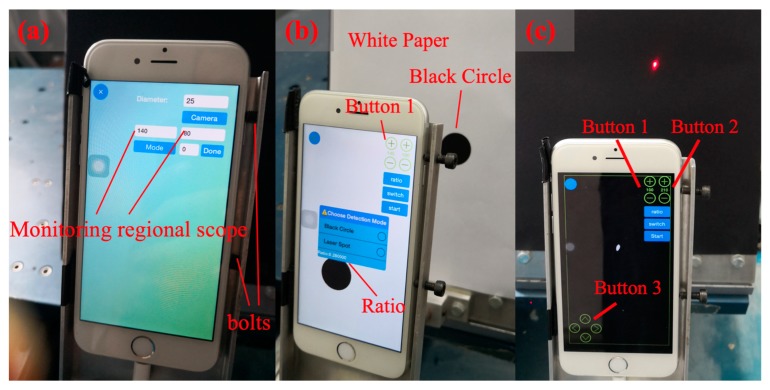
(**a**) Initial setting interface; (**b**) Calibration interface; (**c**) Monitoring interface.

D-Viewer is a smartphone app to implement displacement monitoring. Before the experiment, the iPhone 6 was fixed on the holder by two bolts, as shown in Figure 4a. The bolts were tightened at the right side. There are a series of processes when using D-Viewer as initial setting phase, calibration phase, and measurement phase. 

In the initial setting phase, the D-Viewer software interface is shown in Figure 4a. First, the “Camera” button was used to choose “rear camera,” and the monitoring regional scope was set 140 by 80. Under this condition, D-Viewer could only scan the pixels within this monitoring region to identify the coordinate of the laser spot. Then, in “Mode”, “Single Spot” was chosen. At last, the “Done” button started calibration phase. 

In the calibration phase, the software interface is shown in Figure 4b. Because the calculated displacement is in pixel unit, it should be converted into physical coordinates. A scaling factor should be used in this conversion, and physical displacement could be calculated by Equation (3).

(3) $$\{\begin{array}{c} SF=\frac{D}{d}\\ D_{real}=D_{pixel}\cdot SF \end{array}$$

where D is the physical distance of a target, d is the pixel distance of a target, SF is the scaling factor, $D_{pixel}$ is pixel displacement, $D_{real}$ is physical displacement. Although the distance between the smartphone and projection plate will influence the pixel size of a target, Equation (3) could eliminate the impact of distance when the physical distance and pixel distance of the target is known. During the operation, a white paper with a black circle (Diameter is 25 mm in the experiment.) was used for calibration. The white paper was placed right at the surface of the projection plate. Press button 1 to adjust the threshold value until the black circle was clearly distinguished from the background. Then, click the “ratio” button, the pixel diameter could be obtained in the App, and the scaling factor can be obtained. After that, choose the “Laser Spot” in the dialog box. 

In the measurement phase, withdraw the calibration paper and start the monitoring interface, as shown in Figure 4c. Adjusting button 2 to change the threshold value until the laser spot shown on the app interface becomes small enough. Then, press the “switch” button and press the “3” button to move the monitoring regional scope. This adjustment should make the laser spot locate at the center of the monitoring regional scope. Finally, click the “start” button to start measurement. 

### 2.4. Reference Sensor

**Figure 5:**
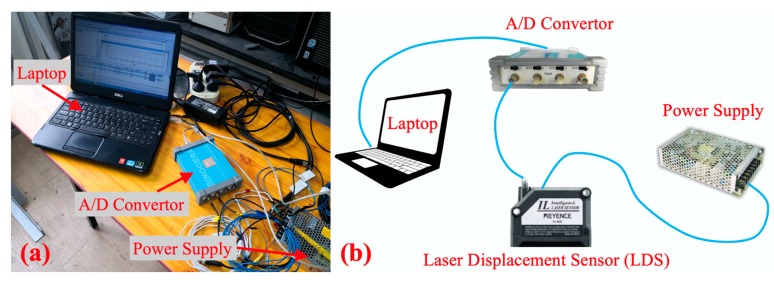
The layout of an LDS monitoring system: (**a**) Data collecting device; (**b**) Instrument connection.

In order to verify the method, two LDS were used as a reference to measure the displacement of the shaking table in two directions. The LDS measurement system is made up of a laptop, an A/D converter, a power supply, and two LDS, as shown in Figure 5. The power supply provides direct current to LDS. Then, the LDS senses the laser waveform change after the laser reflected by the measured object, and this signal is converted from an analog signal to a digital signal by the A/D convertor. Finally, the digital signal is recorded by software on a laptop. 

In Table 1, the details of the instruments’ parameters of the LDS measurement system are listed. 

## 3. Results

Three kinds of experiments—sinusoidal excitation tests, seismic wave excitation test, and night test—were carried out to verify the method. The first two experiments were carried out by adjusting the input wave of a shaking table. The objective of these two kinds of experiments is to verify the feasibility of the method. Meanwhile, the night test was carried out by controlling the light in the laboratory. The objective of the night test is to ensure the image processing method proposed before is stable and robustness in a bad illumination condition. This section will show the results of all the experiments.

### 3.1. Sinusoidal Excitation Tests 

The sinusoidal oscillation is simple, and it could be used to test the method. Therefore, the sinusoidal oscillation with different amplitudes and frequencies were inputted into the shaking table. The experiment scheme of the shaking table test under sinusoidal oscillation is shown in Table 2. Prior to the bidirectional vibration, the unidirectional vibration was carried out in experiments #1 to #6. 

In order to test monitoring results, the data acquired by the smartphone were compared with LDS. Because the data acquisition system is not the same, the time lag existed between two methods. During the data processing, the time axes of two kinds of data were moved back or forward to ensure the peak value would happen at the same time. 

**Figure 6:**
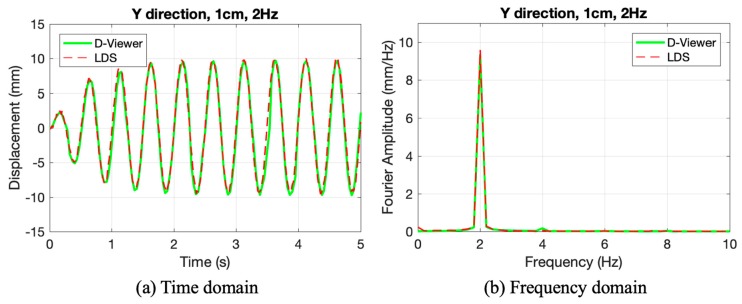
The results of the experiments #7: (**a**) Time domain; (**b**) Frequency domain.

**Figure A1:**
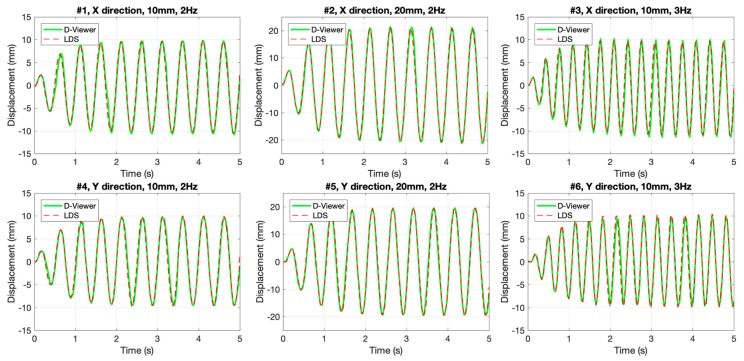
Experiment results of #1 to #6.

**Figure A2:**
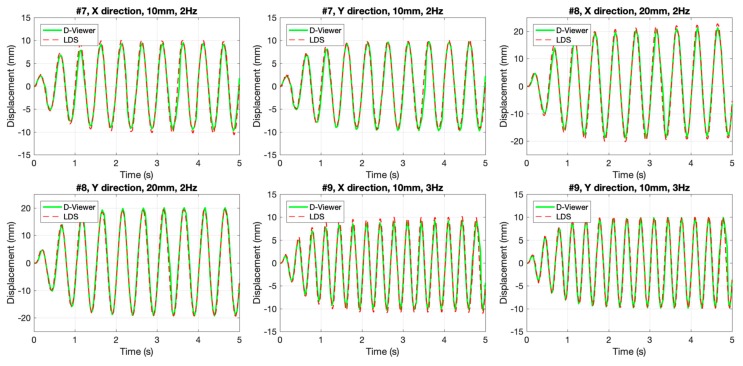
Experiment results of #7 to #9.

Because the results of each test are similar, only part of the data was plotted in Figure 6a. The time-domain monitoring results of rest experiments can be found in Figure A1 and Figure A2 in Appendix A. It is clear that the smartphone agreed well with LDS in two directions in the time history, except for some small deviations at the wave crest, and the error analysis will be discussed later. 

In the experiments, the laser device should be adjusted to ensure that the laser becomes convergent and forms a small laser spot on the projection plate. If the laser is divergent, the laser spot will be very big. A big laser spot will cause the image processing inaccuracy. The result showed the laser device could be competent in the full-scale tests with story-height of 3000 mm. 

Except for the displacement time history curve, the dynamic characteristic is also compared. The data represented in the frequency domain can represent the dynamic characteristics of the vibration. The Fourier amplitude of #7 in Figure 6b shows that the smartphone exhibits excellent agreements with LDS in the frequency domain, the main frequency of 2 Hz of the sinusoidal wave could be detected by smartphone and LDS. With the frequency of 3 Hz carried out in the experiments, the smartphone showed the ability to monitor the fundamental frequency of the most common buildings [2]. In a field test, it has practical significance to measure the fundamental frequency change to assess the building. 

From these tests, the smartphone showed good agreement with LDS with different amplitudes and frequency. As a result, this method could measure the two-direction displacements simultaneously with enough accuracy. 

### 3.2. Seismic Wave Excitation Test

In order to simulate the real building vibration in an earthquake, two seismic waves, El Centro and Northridge, were used as inputs. The corresponding experiment scheme parameters were listed in Table 3. 

**Figure 7:**
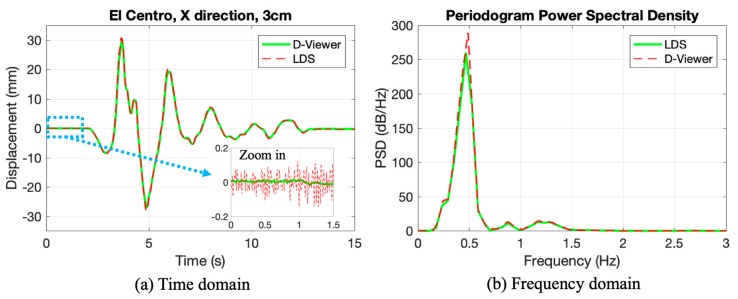
The results of experiments #12: (**a**) Time domain; (**b**) Frequency domain.

**Figure A3:**
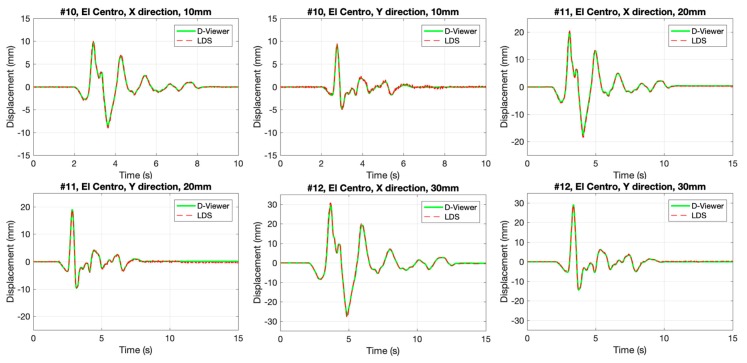
Experiment results of #10 to #12.

**Figure A4:**
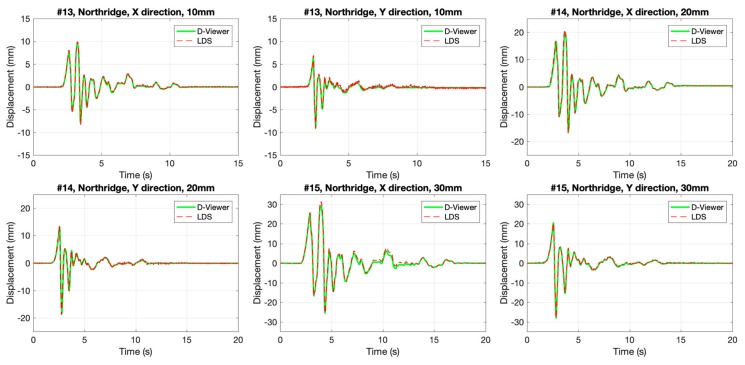
Experiment results of #13 to #15.

The time-domain monitoring results of the seismic wave excitation test are shown in Figure A3 and Figure A4 in Appendix A. There is only one of those results taken as an example, and the results of #12 are shown in Figure 7a in the time domain. In the whole time history, the smartphone is in concordance with LDS. The measuring range of the smartphone could reach 30 mm. Apart from these, local details can be found in the zoomed-in picture. It was observed that the smartphone had a lower fluctuation than LDS, which means the smartphone is more accurate in terms of measurement. The discussion of accuracy and error is in Section 3.5. 

Besides, Figure 7b shows the periodogram power spectral density (PSD) in the frequency domain, and the area between the PSD and the horizontal axis equals to the average power of the stochastic process [32]. Compared with sinusoidal excitation, there are more frequency components in a seismic wave. Although that small difference exists in periodogram PSD, the main frequencies of the two methods are the same. Furthermore, other higher frequency components could also be detected by the smartphone, and the average powers of them are the same as LDS. As a result, the smartphone is in good agreement with the LDS in the frequency domain.

From the results conducted in the seismic wave excitation, it could be concluded that the results of the smartphone aligned well with LDS in monitoring, both in the time domain and in the frequency domain. After the verification under seismic excitation in a real story height, the proposed method is competent in real seismic events monitoring. It could be used to measure the elastic and inelastic response of a building structure. If a building only had an elastic response, a displacement time-history curve would be useful to assess the building. When a building experienced material nonlinearity, both the displacement time-history curve and residual displacement would be significant in the assessment.

### 3.3. Night Test

The professional instrument like PT arrays [19] and DDPS [23] can sense the laser light intensity directly and output the corresponding voltage. Unlike these instruments, the essence of the proposed method is image processing. One of the most important influencing factors in image processing is illumination because the illumination change will cause many image processing algorithms to have poor robustness [17,33,34]. Under a bad illumination environment or no light environment, these methods will have noise in results, or no results would be obtained. As a consequence, the bad results caused by poor robustness should be avoided. 

When a smartphone records videos of the laser movement, the recorded video turned into a series of frames that need to distinguish the laser spot from the black background. Moreover, experiments should verify that the image processing method is robust at night. The illumination difference between daytime and night is the biggest, if the smartphone could be competent at night with the same App settings, it could fulfill continuous measurement. Therefore, a series of experiments were conducted to test smartphones with the same initial setting at night, as shown in Table 4. 

**Figure 8:**
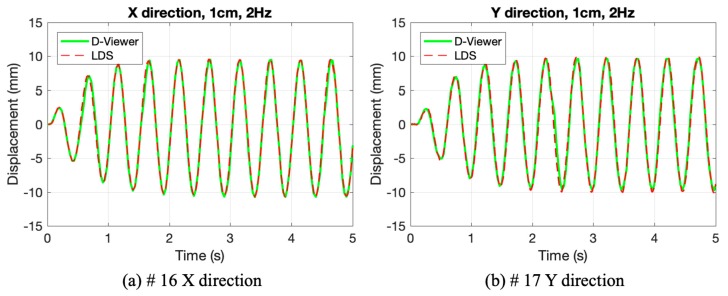
The results of the experiments #16 and #17: (**a**) #16 X direction; (**b**) #17 Y direction.

**Figure A5:**
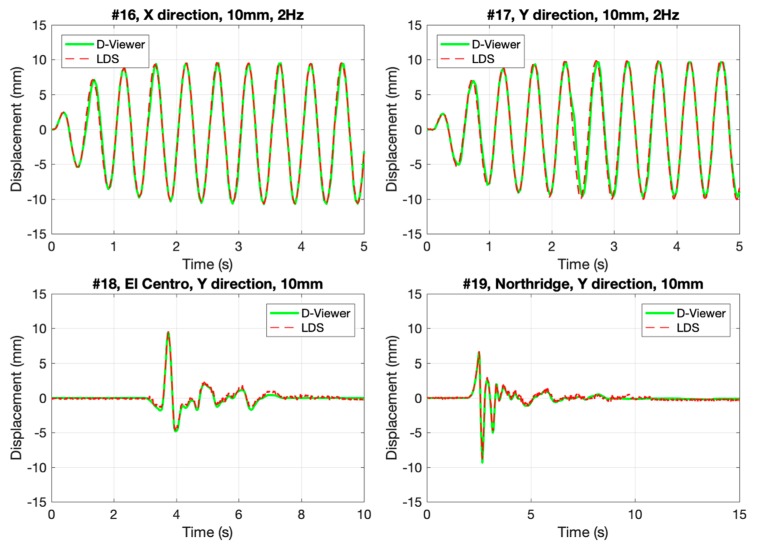
Experiment results of #16 to #19.

In Figure 8, the smartphone agrees well with LDS, and it shows good robustness at night. As a result, after the initial settings in D-Viewer app, this method could be used in continuous measurement no matter in daylight or darkness. The time-domain monitoring results of #18 and #19 can be found in Figure A5 in Appendix A, which also shows good results with poor illumination.

### 3.4. Error Analysis

**Figure 9:**
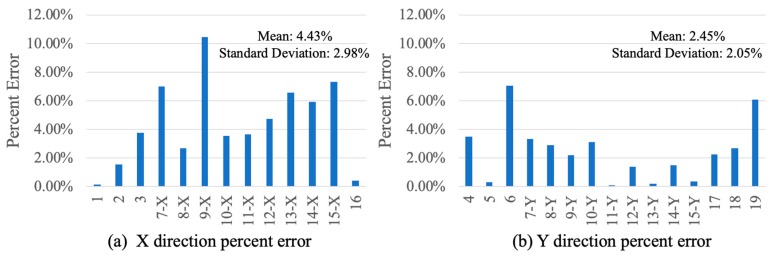
Percent error of two directions: (**a**) X direction; (**b**) Y direction.

Although the monitoring results show that the D-Viewer aligns well with LDS, a small deviation still exists at the maximum or minimum displacement value, which will be discussed in this section. Because displacement amplitudes were different between each experiment, percent error was used to calculate the deviation at the first wave crest of each test. The percent error is defined as the ratio of relative error to the results of the LDS. As shown in Figure 9, the mean value of percent error of all the experiments in Appendix A is 3.37%, with a standard deviation of 2.67%. However, the mean value and standard deviation in the two directions are not the same. The mean value of the percent error in the Y direction is almost half of the X direction. The reason for this might be the real displacement in the X direction is almost half (cos(β)=cos(58°)≈0.53) of displacement on the projection plate in the Y direction, and the relative error is also almost half. 

**Figure 10:**
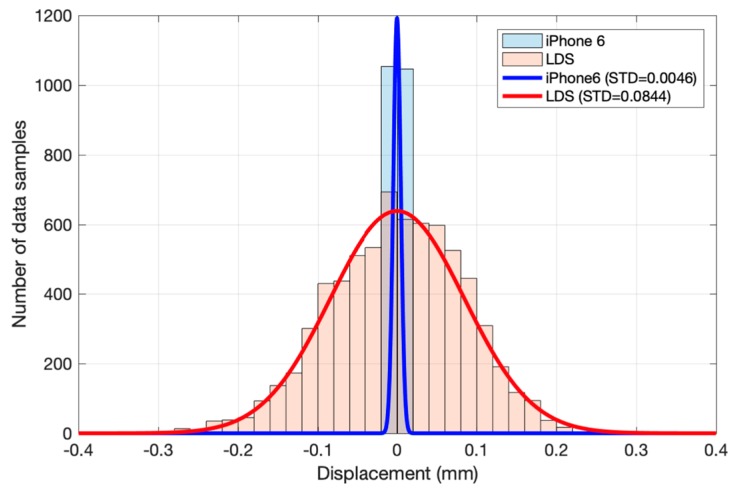
Error distribution of the 70-s data. (The continuous curves have been amplified many times.).

In addition to the errors at the maximum value, errors at a stable period around 0 mm were also evaluated [35]. The zoomed-in picture in Figure 7 has already exhibited a small variation of the smartphone. In this part, to describe the error quantitatively, the monitoring data of a period 70 s were used to evaluate the accuracy of the method. Figure 10 shows that the error approximately follows a normal distribution. Therefore, the normal distribution is used to fit the data. The mean values of the two methods are $-2.7157\times 10^{-19}$ mm (iPhone 6) and $-2.3242\times 10^{-17}$ mm (LDS), respectively. The value is so small that it can be recognized as 0 mm. In addition, the standard deviations (STD) of two methods are 0.0046 mm (iPhone 6) and 0.0844 mm (LDS), respectively. These values were used to draw two continuous normal distribution curves, as shown in Figure 10 in different colours. With smaller STD value, the results of the iPhone 6 are more concentrated, which means iPhone 6 has lower fluctuations in monitoring.

STD is a widely used parameter in statistics. In a normal distribution, the probability of data locates in the interval [μ−3σ,μ+3σ] is 0.9974, where μ is mean, and σ is STD. It will be a rare event that the value locates outside this interval. As a result, in one measurement event, the results of the iPhone 6 have fluctuations, but they are considered in the interval [−0.0138 mm,+0.0138 mm]. This interval length is 0.0276 mm. Because the measurement fluctuation range of the iPhone 6 is 0.0276 mm, it could be regarded as the minimum variance that can be detected by the method. In other words, 0.0276 mm is the accuracy of the method. 

The higher accuracy around 0 mm is meaningful. Because the structure stiffness is high at an earlier stage of seismic damage, a small variation in deformation will lead to a significant change in the internal force. In contrast, when the inter force reaches yield stress, a small variation in force will cause a significant change in deformation [36]. As a result, the high accuracy in the early interstory drift monitoring is meaningful. 

From the error at the wave crest and around 0 mm, it could be concluded that the error is in proportion to monitoring value. Because the monitoring results were identified through multiplying pixel displacement by the scaling factor, the error will be more significant at the large amplitudes. As a result, the percent error was used to describe the error. However, LDS is not designed for interstory drift monitoring—the proposed method should be compared with other methods mentioned in reference to validate its performance instead of LDS, and this will be discussed in the next section.

### 3.5. Sensor Comparison

In order to explain the performance of the method in this study better, it should be compared with other methods. The parameters of other methods mentioned in the introduction are listed in Table 5. In fact, the ways of error representation are different in different reference papers, so the definition should be discussed. In Table 5, precision means the resolution of the method. In other words, it means the minimum variance that can be detected by the method. Percent error means a ratio of absolute error to the reference. Because the measurement amplitudes are different in each test, percent error is a proper parameter to compare the error. The story height no less than 3000 mm means this method has been tested in full-scale tests, which is meaningful in practice. 

The advantage and disadvantages of each method are listed below. The method in paper [15] will be a good choice for rapid residual displacement measurement after seismic events, while it is a static measurement method rather than continuous measurement. The method in paper [16] could describe the deformation condition along a column. However, it needs 8 MEMS inclinometers in one column, thus the cost will increase. The method in paper [17] is easy to handle and has good accuracy, but it needs normal light and has a low sampling rate. The following methods in papers [19,20,21] have high accuracy and high sampling rates, and they have been verified in full-scale tests. Although the method in paper [22] is simple, it has not been verified in full-scale tests—only in the test frame with a story height 200 mm. The method proposed in paper [23] has good performance in full-scale experiments, except for relatively low precision. The advantage of this method is that it contains a full methodology of interstory drift monitoring, including a correction caused by rotation and acceleration calculation. However, this method can only measure unidirectional displacement. 

By contrast, the method proposed in this study shows good accuracy in both precision and percent error. This method had also been tested in full-scale tests. However, the disadvantage of this method is the low sampling rate, which needs to be improved in the future. Due to the popularization of the smartphone, this method has a relatively low cost. 

## 4. Discussion

In the practice of SHM, interstory drift is often acquired by double numerical integration of acceleration data, which will bring error in the results. This paper proposes a method using a smartphone and a laser device to fulfill interstory drift monitoring. This method was verified to be competent in continuous measurement with enough precision. 

After the experiments, some conclusions could be drawn as follows:

1. Compared with the LDS, the proposed method performed well in sinusoidal oscillation, seismic wave vibration, and a night test with a real story height 3000 mm. The monitoring results showed that the smartphone coincided with LDS in time history and frequency domain. The good results of the night test show that the image processing method is robust at night so that the method could fulfill continuous measurement.
2. From the results of all experiments, the average percent error of the smartphone is 3.37%, with a standard deviation of 2.67%. In the stationary period around 0 mm, the standard deviation is 0.0046 mm, which is much smaller than LDS (0.0844 mm). This means the proposed method has a smaller deviation than LDS. The accuracy of the method is 0.0276 mm. These parameters mean the proposed method is competent in interstory drift monitoring with only marginal error.
3. Compared with other similar interstory drift monitoring methods, the method has advantages in terms of precision (accuracy is 0.0276 mm, percent error is 3.37%) and cost, and it has been tested in 19 groups of full-scale experiments. The disadvantage of the method is the low sampling rate of 30 Hz, which needs to be improved in future work.

In conclusion, the proposed method could satisfy the demand of interstory drift monitoring continuously, no matter the illumination conditions. With the popularization and low cost of smartphones, this method could be widely used in monitoring the interstory drift of buildings. When an earthquake strikes a building, smartphones could record the interstory drift time history, which will be useful for engineers to evaluate the damage level of buildings. 

Future extension of the presented research is to develop this app to fulfill higher sampling rate monitoring without changing the focal length. With the development of smartphone hardware, new types of smartphones could record video with 60 frames per second or higher. As a result, it should be verified that the monitoring will be stable at higher sampling rates, and the processing speed of smartphone CPU and storage could fulfill real-time calculation. 

Another extension is to take inclination into account [1,20,23]. Use two or more laser devices to fulfill inclination measurement. Then, the method could monitor the inclination and interstory drift simultaneously with more laser devices and new image processing methods. This inclination could also be used to correct the interstory drift when the floor inclination occurs. 

## Figures and Tables

**Table 1:** The instruments of the LDS measurement system.

Instruments	LDS	A/D Converter	Power Supply	Laptop
Maker	Keyence	Sinocera Piezotronics Inc.	Mean Well	Dell
Model	IL-300	YE6231	NES-100-12	Inspiron 3420
Supplement	Measurement range: 160–450 mm	Signal to noise ratio: 80 dB; 4 channels	Output voltage: 12 V; Direct current	CPU: i5-3210M; RAM: 4G; System: Window 7

**Table 2:** Experiment scheme under sinusoidal oscillation.

Test Number	Direction	Amplitude (mm)	Frequency (Hz)
#1	X	10	2
# 2	X	20	2
# 3	X	10	3
# 4	Y	10	2
# 5	Y	20	2
# 6	Y	10	3
# 7	X, Y	10	2
# 8	X, Y	20	2
# 9	X, Y	10	3

**Table 3:** Experiment scheme under seismic wave excitation.

Test Number	Seismic Wave	Direction	Amplitude (mm)
#10	El Centro	X, Y	10
#11	El Centro	X, Y	20
#12	El Centro	X, Y	30
#13	Northridge	X, Y	10
#14	Northridge	X, Y	20
#15	Northridge	X, Y	30

**Table 4:** Experiment scheme in night test.

Test Number	Waveform	Direction	Amplitude (mm)
#16	Sinusoidal wave	X	10
#17	Sinusoidal wave	Y	10
#18	El Centro	Y	10
#19	Northridge	Y	10

**Table 5:** The parameters of different methods.

Method	Precision (mm)	Percent Error (%)	Story Height (mm)	Measurement Range (mm)	Maximum Sampling Rate (Hz)
Dai [15]	-- ^1^	9.44 ^2^	--	--	Static measurement
Hou [16]	--	7.60	1600	--	--
Li [17]	0.1664	3.09	3000	±200	30
Kanekawa [19]	0.1100	--	8900	±40	200
Matsuya [20]	0.1500	--	3500	±30	--
Matsuya [21]	0.0600	--	3500	±50	1000
Islam [22]	--	10.16 ^2^	200	--	--
McCallen [23]	1.0000	--	3000	±90	384
This study	0.0276	3.37	3000	±60	30

^1^ This symbol means the corresponding parameter is not mentioned in the reference paper. ^2^ This parameter is calculated by the data shown in the reference paper.

## Appendix A

Only parts of the experiment results are shown in the main text for simplicity. All of the experiment results are shown in this appendix as follows (see Figure A1, Figure A2, Figure A3, Figure A4 and Figure A5).
